# Wake up and smell the conflict: odour signals in female competition

**DOI:** 10.1098/rstb.2013.0082

**Published:** 2013-12-05

**Authors:** Paula Stockley, Lisa Bottell, Jane L. Hurst

**Affiliations:** Mammalian Behaviour and Evolution Group, Institute of Integrative Biology, University of Liverpool, Leahurst Campus, Chester High Road, Neston CH64 7TE, UK

**Keywords:** chemical communication, competitive signalling, major urinary proteins, *Mus musculus domesticus*, sexual selection, social competition

## Abstract

Odour signals used in competitive and aggressive interactions between males are well studied in the context of sexual selection. By contrast, relatively little is known about comparable signals used by females, despite current interest in the evolution of female ornaments and weaponry. Available evidence suggests that odour signals are important in competitive interactions between female mammals, with reductions or reversals of male-biased sexual dimorphism in signalling where female competition is intense. Scent marking is often associated with conflict between females over access to resources or reproductive opportunities. Female scent marks may therefore provide reliable signals of competitive ability that could be used both by competitors and potential mates. Consistent with this hypothesis, we report that aggressive behaviour of female house mice is correlated with the amount of major urinary protein (MUP) excreted in their urine, a polymorphic set of proteins that are used in scent mark signalling. Under semi-natural conditions, females with high MUP output are more likely to produce offspring sired by males that have high reproductive success, and less likely to produce offspring by multiple different sires, suggesting that females with strong MUP signals are monopolized by males of particularly high quality. We conclude that odour signals are worthy of more detailed investigation as mediators of female competition.

## 1. Introduction

Darwin [1] recognized the role of odours in sexual selection, noting that specialized scent glands of mammals are often sexually dimorphic, and that ‘the development of these organs is intelligible through sexual selection, if the most odoriferous males are the most successful in winning the females, and in leaving offspring to inherit their gradually-perfected glands and odours’ (p. 530). In support of Darwin's ideas, male-biased sexual dimorphism is often reported with respect to the presence, size and elaboration of specialized mammalian scent glands [2–4], as well the intensity or frequency of scent marking behaviour [2,3,5–9], and the complexity of odour signals [10–13]. Territorial or dominant males typically scent mark at high rates, counter-marking odours of competitors (either over-marking the competitor's scent or increasing the rate of their own scent marking in the immediate vicinity). The result is that the individually recognizable odour of a dominant male predominates within a defended area [3,14,15]. Because scent marks remain in the environment, they are available for both challenge and inspection at any time by other animals visiting the site, even when the owner is elsewhere. Thus, the spatial and temporal pattern of scent marks deposited by males provides a particularly reliable broadcast signal of their competitive ability in dominating and defending a territory, which can function both in intrasexual and intersexual selection [15–18].

In contrast with males, relatively little is known concerning the role of odour signals in female competition. Here, we review current literature on the scent marking behaviour and odour signals of female mammals, with emphasis on roles in competition for resources and reproductive opportunities, and on reproductive advertisement. We also present new data for house mice (*Mus musculus domesticus*) suggesting that major urinary proteins (MUPs) used in odour communication may provide a signal of female competitive ability that reflects their ability to secure resources and high-quality mates.

## 2. Do females also use odour signals in a competitive context?

Forty years since Johnson [3, p. 527] remarked *‘*there seems to be a particular lack of data on the scent marking behaviour of females’, the study of female odour signals is still relatively neglected (e.g. [7,12,19–28]). This may be due partly to low expectation that competitive signalling is important for females [29–32], but also because odour signals are easily overlooked. Visually conspicuous signals or ornaments expressed by females are relatively well known by comparison, but until recently were often regarded as a correlated response to sexual selection acting on males [29]. However, it is now more widely recognized that such signals can evolve independently of selection on males [30], that they may signal female quality [33–37], and that males may prefer females with larger or more conspicuous signals [32,36,38]. Competition for resources has also been linked to visually conspicuous female signals, indicating that these may function primarily in communication with other females [31,32,39,40]. Hence it seems likely that odour signals used by females in species that rely on chemical communication will serve similar functions to those of visually conspicuous signals expressed by females of other taxa.

Although early studies of sexual dimorphism in olfactory communication placed strong emphasis on greater investment by males [1,4], examples can also be found in which adult females invest heavily in odour signals, in some cases more so than males. Male-biased dimorphism in scent marking is typically reduced in monogamous species [41] and examples of greater female investment are found among species with high levels of offspring care by males. Rates of scent marking are strongly female biased among callitrichid primates in which male care exceeds that of females, with significantly more frequent, intense and diverse female marking behaviour [5,26,42,43]. Female scent glands in such species are also much larger than those of males [26,42]. Female bush dogs (*Speothos venaticus*), not males, perform elevated handstand urine marking as well as several other scent marking behaviours shown less often or never by males [21]. In these examples, greater investment in olfactory signalling by females is interpreted in relation to intrasexual competition for high levels of male offspring care—signalling both to attract mates and to exclude competition from other females [5,21,26]. Notably, as for males, the same signals may be used by females in both contexts. Assistance with offspring care in these species could also offset increased costs of signalling by females that might otherwise limit their parental investment. A reversal of the dimorphism in scent signal complexity, which is usually male biased, is also found in some species where females exhibit relatively high levels of aggression and are socially dominant over males. Among female-dominant lemurs of the genus *Eulemur*, female glandular secretions are more chemically complex than those of males but this is not the case among co-dominant species. Greater morphological elaboration of anogenital glands, with increased perianal folds, is also found in females of *Eulemur* species compared to males [13].

Studies of female counter-marking behaviour are still rare, but cases of relatively high female counter marking to scents from the same sex have been reported. Female meadow voles (*Microtus pennsylvanicus*) deposit significantly more counter marks than males when placed in experimental arenas previously scent marked by a same-sex conspecific [44]. However, the functional significance of this behaviour is unclear, and female marking behaviour of rodents does not always involve direct counter marking in response to same-sex conspecifics [19,23]. Although not female biased, a relatively low sex bias in counter-marking behaviour has been reported for banded mongooses (*Mungos mungo*) under natural conditions [27]. In a field experiment using translocated faecal scent marks, females almost exclusively counter-marked scent marks of other females, placing their own marks on top of the original marks. In this case, high rates of female counter marking are linked to intense competition between females for breeding opportunities [27].

The examples described above illustrate that female investment in odour communication can be significant. Importantly, female scent marking behaviour may also be under-reported if it occurs in different contexts to scent marking behaviour of males. For example, females may scent mark more in social contexts rather than spontaneously, at specific stages of the reproductive cycle or in response to odour cues of the reproductive cycle of other females ([45], and see below). Scent marking and associated investment in odour signals may also be more variable among females than among males. Female Mongolian gerbils (*Meriones unguiculatus*) mark at high male-like rates only during lactation [46]; at other stages of the reproductive cycle marking frequency shows a skewed distribution with mostly low scores and some much higher markers, compared to a normal distribution of marking frequencies for males [47]. Comparing average investment of males and females in such cases may be misleading.

## 3. Female odour signals in competition for resources and reproductive opportunities

Female competition is commonly linked to resources such as food, nest sites, water or helpers needed for successful reproduction [48–50]. In some cases, valuable resources are scent marked directly [51], or marks may be deposited in the immediate vicinity while using a resource, which may signal priority of use [52]. As described below, odours can also have a range of social functions in the context of dominance relationships determining priority of access to resources or reproductive opportunity, as well as intergroup resource defence and territoriality.

### (a) Dominance and reproductive suppression

Females of social species often establish dominance relationships that can determine access to limited resources and hence influence reproductive opportunities or relative success [50]. As such, odour signals may have important functions in advertizing and maintaining social or reproductive dominance [2,26]. Consistent with this hypothesis, higher rates of scent marking [27,53–55], counter marking [24] and larger or more elaborate scent glands [55,56] are often reported for dominant females compared to subordinates. For example, high-ranking ring-tailed lemurs (*Lemur catta*) counter mark the genital marks of other females at higher rates than do low-ranking females [57]. However, different patterns of scent marking are also found in relation to social dominance, such as high rates of anal marking performed at the border of territories by subordinate yellow mongooses (*Cynictis penicillata*), in areas never visited by dominant females [58], and high rates of scent marking by reproductively subordinate common marmosets (*Callithrix jacchus*) during intergroup encounters [51]. Heymann [26] also notes that evidence to support a function of scent marking in the direct regulation of social status is mixed, and that female scent marking may instead function in signalling individual quality, both to other females and to potential mates (see also below).

Both scent marking and reproductive activity of subordinate females are suppressed in the presence of dominant breeding females in many callitrichid primates [5,56,59–61], and odours from dominant females are implicated in facilitating this suppression [62–64]. Nonetheless, interpreting the function of female scent marks as a signal to inhibit the reproduction of others is not straightforward. In particular, it is puzzling why subordinates should sniff scent marks that will result in reduced fitness via physiological manipulation [5]; further, the dominant female will presumably have little control over who actually receives the signal if scents are left in the environment [65]. There is also evidence that the response of subordinates to odour signals of dominant females is dependent on the individual identity of the signaller [66]. If reproductively suppressed common marmosets are isolated from their social group, resumption of the ovarian cycle is delayed by continued exposure to the odour of a dominant female that is familiar [64], but not in response to the odour of a dominant female that is unfamiliar [66]. Hence reproductive suppression of subordinates is not due simply to a pheromone produced by dominant females. Also, prolonged suppression is not sustained by odour exposure alone, but requires direct interaction with the dominant female [66]. If odour signals of dominant females function as a threat of direct aggression, reproductive suppression among subordinates may instead be self-imposed [2,5,66]. By suppressing their own reproduction, subordinate females may thus avoid costs of aggression, including potential eviction from the social group or infanticide [66,67]. More generally, female mammals may be predisposed to resolve intense conflict with reproductive suppression due to the high reproductive investment associated with gestation and lactation [68,69].

### (b) Territoriality and intergroup aggression

Scent marking is often associated with intolerance of conspecifics, and many species mark more frequently after encounters with those from outside their home range or territory, particularly with members of their own sex [2,6,7,9,15,18,19,70–73]. Among social or pair-living species, the edges or borders of home ranges that overlap with neighbours may be scent marked intensively by both sexes ([71,74–76] but see [77]), and particular attention is paid to scent marks from neighbours. In field experiments where conspecific scent marks were translocated, banded mongooses responded more strongly to scents from neighbouring groups than from strangers [75]. Monogamous aardwolves (*Proteles cristatus*) of both sexes responded with increased scent marking and went directly to the border of the neighbour whose scent they had encountered [70]. Alternatively, aggression may be relatively low between neighbours, termed the ‘dear enemy effect’, but greater towards dispersing or itinerant individuals that pose a greater competitive threat, especially during the breeding season, as suggested for European badgers (*Meles meles*) [78]. Notably, members of the same social group may have a distinctive shared group odour [79–81], and use the same communal marking sites (e.g. [3,21]). Such odours may be beneficial in promoting cohesive relations among group members [21,81], or in territoriality by establishing an asymmetry in resource defence potential between residents whose scent will match communal odours within the territory and intruders [14,18,79]. However, evidence for discrimination on the basis of group odour in territorial defence is currently mixed and limited to relatively few species [75].

Scent marking can also be important for resource defence and territoriality among solitary species [18]. Vaginal scent marks appear to function in the spacing behaviour of female Syrian hamsters (*Mesocricetus auratus*), as females avoid areas scent marked by other females under laboratory conditions [22]. In the solitary honey badger (*Mellivora capensis*), token urination (small amounts of urine dribbled from a squatting position) was mainly observed in females and also appears to function in spatio-temporal separation, showing no seasonal variation or changes in relation to the oestrus cycle [82]. By contrast, normally solitary female bank voles (*Myodes glareolus*) kept together in large complex enclosures rarely scent marked until they were pregnant or lactating, when scent marking with urine was often associated with agonistic interactions. In this case, the timing of increased female aggression and scent marking is consistent with maternal aggression and nest defence [23].

## 4. Reproductive advertisement and competition for mates: signals of female quality?

Advertisement of fertility or sexual receptivity is a widely acknowledged function of female scent marking behaviour [2,3,83], and numerous sources of olfactory cues can convey information concerning oestrous state, including urine and sebaceous gland secretions [84–87]. Female scent glands often secrete most during the mating season [3], and females may also scent mark with increased frequency, or exclusively, during periods of sexual receptivity [3,20,24,25,83,84,88–93]. Even when there is no cyclical variation in female scent marking rates, male sensitivity to qualitative changes in female scent is still reported [94,95]. The urine of female laboratory mice, for example, contains sex-specific sulfated steroid hormones that are detected both by other females and by male mice through specific receptors in the vomeronasal olfactory subsystem, potentially providing detailed information about the female's physiological state on contact with the scent [96–98].

Self-advertisement via female scent marking is likely to facilitate sexual attraction of males, which may be of particular benefit in solitary species. However, a function of attracting males is also commonly suggested for social or pair-living mammals, particularly in relation to scent marks deposited at the borders of home ranges that may attract males from neighbouring groups or territories [27,51,58,99]. Male aardwolves reportedly use scent marks deposited by neighbouring females on territory borders to time visits in pursuit of extra-pair copulations [99]. Another suggested function of scent marking by oestrous females is to stimulate male competition and increase the probability of mating with high-quality mates [100]. Odours from vaginal or other secretions and excretory products may combine to provide very precise information about female reproductive state, leading to a graded pattern of attractiveness [101]. Such precise information about female fertility in turn may allow competitively successful or dominant males to monopolize females at an optimal time for achieving fertilization success, with potential fitness benefits for females (cf. the graded signal hypothesis for primate sexual swellings [102]).

Although a function of attracting males during periods of sexual receptivity is widely accepted in relation to female scent marking behaviour, the role of female odour signals in competition for mates has received less attention [103]. Females may compete for males in order to gain access to territorial resources or offspring care that will directly benefit their reproductive success, or to gain indirect genetic benefits for their offspring, particularly where preferred males are at risk of becoming sperm depleted [50]. Notwithstanding theoretical issues relating to potential trade-offs with offspring production [104–106], odour signals often appear costly and might function as reliable indicators of female quality, analogous to condition-dependent signals more typically hypothesized to advertise heritable fitness in males ([107,108], but see [109]). Alternatively, if females benefit from mating multiply but males are sperm limited and choosy [110–111], then exaggerated signals of female fertility or receptivity might instead result from antagonistic coevolution with male resistance (or ‘chase-away’ selection, [112]), resulting from sexual conflict over female remating rates.

Competition for mates can be exacerbated if multiple females become sexually receptive simultaneously, as occurs in banded mongooses [28]. In field experiments, scent marks from oestrous females were more likely to be counter marked by other females than scents from females not in oestrus, while oestrous females themselves showed a striking increase in counter marking, suggesting intrasexual competition to be particularly intense during periods of sexual receptivity [27]. However, Jordan *et al.* [28] found only limited evidence that counter marking by female banded mongooses functions in competition for mates. Although females with high rates of counter marking were more likely to be guarded by males in better condition, high counter-marking females were not mate guarded for longer than those with low rates. Hence, although a function of reproductive advertisement is commonly assumed for female scent marking, it is important to consider that increased scent marking during oestrus might also function in communication with other females. Odour communication between females is suggested to reduce competition for preferred males by facilitating avoidance of oestrus synchrony in ring-tailed lemurs [113]. A competitive element to such communication might also result if reproductive advantages can be gained by monitoring the reproductive state of other females [57].

## 5. Competitive signalling in house mice: a role for major urinary proteins in female competition?

### (a) The use of urine for competitive signalling in house mice

House mice are an important model species for the study of scent communication and have been the subject of detailed biochemical and behavioural analyses of urinary chemosignals used in competitive signalling among males [6,7,10,15,114–118]. Dominant male house mice are territorial, depositing urine marks extensively around their territories, and counter mark odours of rival males by increasing their own marking rate in the immediate vicinity [6,15]. These competitive scent marks influence female preference between males, with a consistent preference for males that counter mark competitive challenges [17,119]. These scent marks contain information about the relative freshness of each male's scent along with the species, sex, social status and individual identity of the owner, encoded by a complex set of androgen-dependent volatiles, urinary metabolites and urinary proteins [15].

Social organization among female house mice is variable but several females usually share the same range and nest sites, often overlapping the territories of several dominant males [120,121]. Competition for reproductive opportunities can be intense in high-density populations, with variation in reproductive success linked to access to resources and variation in the quality of nest sites [121]. Along with dominant males, some female mice also contribute to territorial defence of resources from invading conspecifics [121]. Female scent communication therefore has likely functions in competition for reproductive opportunities and for resources, and Hurst [7] found evidence that urine marking plays an important role in communication between females. Notably, resident breeding females showed a strong counter-marking response, especially towards neighbour urine, and Hurst [114] found that breeding females counter-marked urine from unfamiliar breeding females and resident subadult females particularly strongly. Female mouse urine also contains pheromones known to inhibit the reproductive physiology of other females under conditions linked to competition for reproductive opportunities [117,122]. While older dominant females continue to breed even in high-density populations, removal of these females leads to earlier sexual maturation and reproduction of younger females [7,123].

### (b) Changes in investment in major urinary proteins

The urine of male mice is characterized by an unusually high concentration of proteins, over 99% of which comprises the MUPs, a group of 18–20 kDa lipocalins [10,15]. Most MUPs involved in mouse chemical signalling are synthesized in the liver for excretion in the urine [15], although some MUPs are produced in other tissues, such as salivary and lachrymal glands, nasal tissues or mammary glands [124]. A notable characteristic is that these proteins have a central calyx that binds small hydrophobic ligands, including several known male volatile pheromones, and facilitates a substantially slowed release of highly volatile ligands from scent marks [15]. Thus, the amount of MUP excreted influences not only the strength of the protein signal itself in the scent mark but also the concentration of many volatiles held and released from scent marks over time. Mouse urinary MUPs are also extremely polymorphic and provide a signal that identifies the individual scent-mark owner [119,125]. Detected through direct contact, the involatile MUPs themselves can stimulate specific behavioural responses such as increased aggression between males [118]. Furthermore, a male-specific MUP named darcin is responsible for female sexual attraction to spend time near male urine and stimulates a remembered preference for its spatial location in both females and competitor males [126,127].

**Figure 1.**
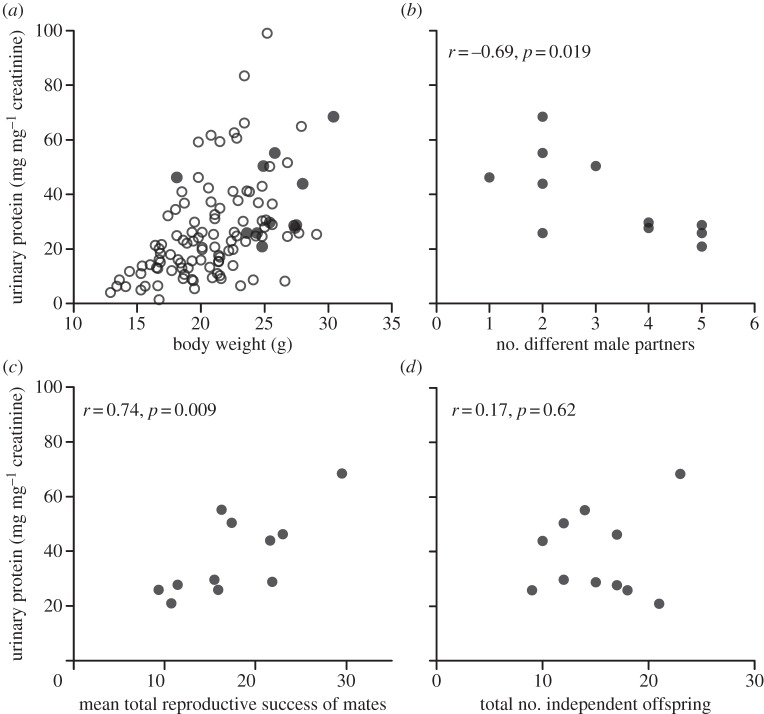
Urinary protein output of female house mice captured from four large semi-natural enclosure populations founded by 33 females and 48 males. Protein concentration is expressed as mg mg^–1^ creatinine to correct for urine dilution [10]. SDS-PAGE confirmed that urinary protein consisted almost entirely of MUPs. (*a*) Urine samples were obtained from *n* = 11 of the founder females (filled circles) aged 23–28 weeks and *n* = 106 female offspring (open circles) aged up to 17 weeks, sampled from a total of *n* = 497 independent offspring captured at the end a four-month experiment where founders were allowed to breed freely to assess inbreeding avoidance (see [130] for full details). Urinary protein concentration did not differ significantly between populations (*F*_3,86_ = 0.12, *p* = 0.95) or between founder females and their adult (more than 18 g) female offspring (*F*_1,86_ = 2.95, *p* = 0.09), but increased significantly with body weight (all females: *r*_117_ = 0.48, *p* < 0.0005; adult females more than 18 g: *r*_94_ = 0.31, *p* = 0.002). Females over 25 g were very likely to be heavily pregnant at sampling. (*b*) Offspring from each female were assigned to separate matings with different males according to parentage analysis using 32 microsatellite markers, offspring weight and capture date (focal females had three successive litters with offspring more than three weeks of age at the time of capture). For each female, the total number of males that sired offspring that survived to independence was summed. Founder females with the highest protein output had the fewest successful male partners. (*c*) For each focal female, we calculated the mean total reproductive success of the males that sired their offspring (the average number of independent offspring sired by each male with any female in the population, weighted by the number of matings with the female). Founder females with higher protein output mated with males that had higher overall mating success. (*d*) There was no evidence that founder females with high urinary protein output produced more offspring that survived to independence. We confirmed that none of these relationships were due to differences between the four populations or to differences in creatinine levels that might reflect differences in urine dilution or in muscle mass between females.

Although studied most extensively in relation to competitive and sexual signalling in males, MUPs are also present in the urine of female house mice. The individual-specific pattern of MUPs expressed by females is as complex as that of males, although females lack expression of a small number of MUPs that are androgen-dependent [10,128]. However, under standard laboratory conditions (single-sex caged housing) female MUP output is relatively low, with females of laboratory strains expressing twofold to eightfold less urinary protein than males of the same strain [129]. However, females captured from the wild appear to have much higher urinary protein concentrations [10]. To examine female investment in MUPs more closely under naturalistic social conditions, we have looked at urine samples from wild house mice living in four large (250 m^2^) outdoor enclosures that were obtained (when practicable) at the end of a four-month experiment that was designed to assess the genetic basis of inbreeding avoidance [130]. This revealed a substantial increase in MUP investment in both sexes under semi-natural compared to laboratory conditions. Male MUP output increased approximately fivefold compared to a typical range of 2.5–15 mg protein per milligram creatinine for laboratory mice [131]. The increase among females was even more dramatic (figure 1*a*). Among adult females (more than 18 g), urinary protein output ranged from 5.5 to 99 mg protein per milligram creatinine (to correct for urine dilution [10]), with a mean of 30.7. This compares to a mean of 3 (range 0.8–6) among laboratory females [129], which is similar to MUP output for wild females housed under laboratory conditions (J. L. Hurst 2009, unpublished observation). An increase in MUP concentration has also been reported for laboratory strains during the early period of sexual receptivity, as predicted if MUPs and/or their bound ligands function in reproductive advertisement to males [132,133]. However, the level of within-individual variation across the oestrous cycle appears to be small compared with the very high level of individual variation in urinary protein output among wild female house mice (J. L. Hurst 2009, unpublished data), and other urinary components are also likely to vary with hormonal status across the oestrous cycle.

### (c) Does major urinary protein output predict female mating and reproductive success?

As surviving independent offspring of the original founding animals in the outdoor enclosures were genotyped to assess parentage and identify mating partners over a four-month period, we are also able to examine the relationship between urinary protein output for the random subset of founding females for which we gained a urine sample (on capture at the end of the four-month period) and measures of reproductive success (figure 1*b–d*). Multiple mating in these populations was very high overall, with 67% of litters sired by more than one male (based on paternity assignment for 193 successful copulations) [130]. However, the urinary protein output of focal female mice was negatively correlated with the number of males that sired their offspring (figure 1*b*), such that females with high urinary protein produced offspring sired by fewer different males. This pattern suggests that females with high urinary protein output are perhaps more likely to be monopolized by high-quality males. Consistent with this hypothesis, we find a positive relationship between the urinary protein output of female mice and the average total reproductive success of the males that sire their offspring (figure 1*c*). By producing offspring sired by males with high overall mating success, females with high urinary protein output may therefore gain heritable fitness benefits for their offspring [50,134]. By contrast, we found no evidence that females with high urinary output produced more surviving offspring (figure 1*d*), perhaps because resources required for successful reproduction were not limiting in the very large outdoor enclosures and all but one founder female produced the maximum number of litters possible over the experimental period with offspring that survived to independence.

### (d) Does competitive social pressure increase major urinary protein output?

Under naturalistic conditions, females undergo competitive and sexual interactions as well as maternal investment (including the defence of pups) that may influence their investment in MUP signalling. Garratt *et al*. [135] compared urinary MUP output among female house mice that were housed under carefully controlled social conditions within laboratory enclosures for a period of 16 weeks to separate the effects of reproduction from the requirement to defend the breeding territory. This showed that breeding females housed with a male increased their investment in urinary MUPs by approximately threefold when they had to defend their territory from a neighbour pair compared to control non-breeding females (housed in single-sex pairs under non-territorial conditions). By contrast, equivalent females in breeding pairs that had no competitive pressure from neighbours did not increase their MUP output significantly compared with control non-breeding females. A very similar pattern of elevated MUP output under competitive breeding conditions was also seen among males [136], indicating that competitive social pressure is an important factor influencing increased investment in MUP signals in both sexes. Thus, breeding alone was not sufficient to explain increased investment in MUP signals, but increased MUP output could relate to defence of resources, competition for sexual partners, parental defence of offspring in the presence of potential danger or a combination of these.

### (e) Does urinary protein investment predict aggressive behaviour of female mice?

If the amount of MUP in female scent marks is involved in signalling female competitive ability, the aggressive or competitive behaviour of female mice should be correlated with their urinary protein output. Results of a behavioural experiment designed to test this prediction under controlled conditions are summarized in table 1. Frequency of aggressive behaviours on meeting an unfamiliar same-sex conspecific was significantly related to the amount of protein in the urine of oestrous female mice, with higher protein (controlling for variation in urine dilution [10]) predicting more frequent aggression (table 1*a*). By contrast, submissive behaviours were not significantly related to urinary protein concentration (table 1*b*). As our previous studies have shown that urinary protein consists almost entirely of MUP among healthy adults of this species, MUP output predicts female aggressiveness towards competitor females.

**Table 1.** Generalized linear mixed models (GLMMs) to investigate if urinary protein output of female house mice predicts total number of (*a*) aggressive or (*b*) submissive behaviours recorded during a 30 min encounter with an unfamiliar female conspecific matched for body size (see the electronic supplementary materials for methodological details and behaviour classifications). GLMMs were used with a logarithm link function and Poisson distribution, fitted using the Laplace approximation to restricted maximum-likelihood estimation (lmer procedure in the lme4 R package, [137]). Female mice (*n* = 48) expressed between 0 and 52 aggressive behaviours (median = 0) and between 0 and 55 submissive behaviours (median = 8) during 30 min encounters. Data for body mass and urinary protein output (corrected for urine dilution [10]) were log-transformed prior to analysis. Urinary protein to creatinine ratio (*n* = 45, mean ± s.e. 6.76 ± 0.54, range 1.28 to 18.95) was not significantly related to body mass (*n* = 48, mean ± s.e. 18.83 ± 0.38 g, range 13.9–24.7 g; linear regression *F*_1,44_ = 1.10, *r*^2^ = 0.02, *p* > 0.30). Experimental pair was included as a random effect. In each model, number of observations (individuals) = 45, and number of groups (pairs) = 23.

fixed effects	coefficient (s.e.)	*z*-value	*p*-value	random effects	variance (s.d.)
(*a*) model for number of aggressive behaviours					
(intercept)	−55.27 (8.90)	−6.21	<0.001	pair	14.86 (3.85)
body mass	40.34 (7.00)	5.84	<0.001		
urinary protein	1.86 (0.76)	2.43	0.015		
(*b*) model for number of submissive behaviours					
(intercept)	22.86 (3.06)	7.47	<0.001	pair	1.51 (1.23)
body mass	−16.23 (2.38)	−6.83	<0.001		
urinary protein	−0.16 (0.30)	−0.54	0.59		

These results suggest a potential role for MUPs as competitive signals used by female house mice. As MUPs bind volatile ligands (see above), increased investment in MUPs may also affect the concentration of bound volatiles in scent marks and the duration over which these are released, although female-specific MUP ligands have not yet been identified. Direct aggression occurs between female mice under natural conditions, albeit at much lower frequency than among males [121,138,139]. Moreover, although aggressive behaviour has been linked to female reproductive state [140], as shown here it is not restricted to pregnant or lactating females [121,141]. Importantly, Hurst [121] found that the expression of aggressive behaviour by female mice in high-density populations was linked to breeding success and access to resources. The most competitively successful females achieved unrestricted access to resources, successfully reared offspring to independence, and were the only females to attack resident territorial males. These females also actively defended resources against invading individuals of either sex. Urinary scent marks of aggressive and competitively successful females may therefore have relatively high MUP content, signalling competitive ability to conspecifics both within and between territories. By providing reliable signals of competitive ability, scent marks might then function ultimately to reduce direct aggression over reproductive opportunities or resources, for example, by serving as a threat to younger or less competitive females within the same social group [67,142], or as a signal to intruders that causes them to retreat when encountering the female signaller [14].

## 6. Conclusion

Despite growing interest in the evolution of female ornaments and weaponry [30–40,69,106], the use of odour signals in female competition is still not well understood. Evidence reviewed here suggests odour signals are often used in competitive and aggressive interactions between female mammals and are likely to have important functions beyond reproductive advertisement. Our data for female house mice also suggest that female scent marks may provide reliable signals of competitive ability or quality that could be used both by competitors and potential mates. We conclude that female odour signals are worthy of more detailed investigation as mediators of female competition.

## Notes

This research adhered to the Association for the Study of Animal Behaviour/Animal Behaviour Society Guidelines for the Use of Animals in Research, the legal requirements of the country in which the work was carried out and all institutional guidelines.

## Data accessibility

Data are available from the Dryad Digital Repository: http://dx.doi.org/10.5061/dryad.fk444.
